# Supplementary material for: Are carbon emissions trading and green financial instruments synergistic? -Comprehensive quantitative research based on content analysis
Source: PLoS One. 2024 Mar 7;19(3):e0298601. doi: 10.1371/journal.pone.0298601 (PMC10919601; doi:10.1371/journal.pone.0298601)
Supplement: S1 Data — (ZIP) [file pone.0298601.s001.zip › Data/地级市Z_F工作报告.zip!/╣π╢1⁄2/╔╟╬▓╩╨/└┤╫╘╣1⁄2╓┌║┼í╛┬φ┐╦╩2╛▌═°í┐_2013.docx]

各位代表：

　　我代表市人民政府向大会作政府工作报告，请予审议，并请政协各位委员和其他列席人员提出意见。

　　一、2012年工作回顾

　　2012年，在省委、省政府及市委的正确领导下，在市人大及其常委会和市政协的监督支持下，市政府坚持以邓小平理论、"三个代表"重要思想和科学发展观为指导，全面实施"四大战略"，加快"八大转型"，攻坚克难，狠抓落实，推进科学跨越发展、建设幸福汕尾，争当促进区域协调发展排头兵，顺利完成了市六届人大一次会议确定的目标任务。

　　（一）经济增长态势良好。

　　2012年全市生产总值610.4亿元，增长13.5%；规模以上工业增加值183.6亿元,增长28.3%；公共财政预算收入41.1亿元，增长25.6%；市区居民人均可支配收入18422元, 增长17%，以上四项指标增速均居全省第一位。固定资产投资391.6亿元，增长18.8%；人均GDP突破2万元大关；农民人均纯收入8569元，增长16%。

　　（二） 转型升级初见成效。

　　产业结构进一步优化。优势传统产业转型升级加快，实施技改创新项目26个，投入技改资金27.2亿元，增长123.2%，10个转型升级示范点企业取得成效。电子信息产业基地初步建立，目前全球最先进的光电嵌入式单片电容屏(OGS)生产线在信利建成投产；市城区"电子信息产业基地"通过省的评审。工业增加值占GDP的43.7%，比2011年提高3.1个百分点。商贸、旅游、金融等现代服务业加快发展。第三产业完成增加值219.1亿元，占GDP的35.9%；社会消费品零售总额424.3亿元，增长11.9%；银行金融机构贷款余额增长21.1%，其中涉农贷款增长32.8%。成功竞得省3亿元旅游扶持资金，红海湾被授予"广东省滨海产业示范园区"；长沙湾度假城、岁宝百货等一批商贸旅游项目加快推进。

　　重点项目拉动明显。红海湾电厂3、4号机组并网发电，创产值超62亿元，脱硫、脱硝系统通过环保部验收；华润海丰电厂厂平工程进展顺利，主厂房第一罐混凝土浇筑；陆丰核电有效推进；甲湖湾电厂获得国家发改委"路条"，厦深铁路汕尾段、潮惠高速汕尾段加快建设，广州至汕尾高铁前期工作有序推进。深汕特别合作区基础设施不断完善，腾讯等34家企业相继入园，预计总投资247亿元。重点项目推动县域经济提速发展，海丰县综合竞争力居全省第十位。

　　"三农"工作成效突出。农业总产值164.4亿元，增长6%；粮食总产量近50万吨，粮食生产实现"九连增"。农业产业化不断推进，新增省、市级农业龙头企业10家、省级现代农业园区2个、省级农业名牌产品5个；农业机械化综合水平达52%；国泰、五丰等农产品加工示范区建设加快。海洋生产总值188亿元，增长15%。新农村建设取得成效，规划建设51个名村、96个示范村；建成农村信息服务站、体验站247个；陆丰市成功竞得2亿元村村通自来水工程示范县建设资金。

　　宜居城市建设加快。完成了《汕尾市综合交通规划》等多项专项规划编制工作；创建市级试点宜居城镇10个、宜居村庄21个、宜居社区17个。城乡交通网络进一步完善，建设公路里程224.3公里。完成了体育路、工业大道南段、腾飞路东段等市政道路建设。生态工程深入实施，完成了12宗病险水库加固工程和16公里"百里海堤爱海工程"建设；全年植树造林42万亩，取得历史性突破，建设城市景观带52公里、沿海防护林带43公里，森林覆盖率达51.9%，成功创建"广东省园林城市"。市生活垃圾无害化处理中心等市政设施建设加快推进，顺利实现节能减排目标。资源环境管理不断加强，处置清理闲置土地、打击非法开采稀土、河沙等矿产资源成效明显。

　　企业不断发展壮大。民营经济实现增加值436.6亿元，增长15.8%，占全市生产总值71.5%；规上工业企业实现利税总额38.5亿元，增长104.7%；利润总额25.2亿元，增长126.3%。新增个体工商户9084户；新增私营企业574家；创办5家研发机构、7家企业研发中心；专利申请量762件，增长120%，增幅居全省第一位。信利光电等一批重点企业正在开展上市前期工作。

　　外向经济稳步发展。进出口28.4亿美元，增长13.6%，增速居全省第三位；实际利用外商直接投资3.45亿美元，增长13.2%。加工贸易加快转型升级，信利半导体、德昌电子等13家企业被认定为"全省加工贸易转型升级示范企业"。口岸开放步伐加快，小漠港、甲湖港正式列入国家"十二五"口岸扩大开放规划。

　　（三）幸福汕尾建设全面推进。

　　社会保障成效明显。新增城镇就业45379人，城镇登记失业率2.6%；农村劳动力转移就业率73.3%，受到省政府的表彰。全市社会养老保险和医疗保险制度实现全覆盖，新农合覆盖率100%。社会救助体系逐步完善，社会福利和慈善事业稳步发展。全面完成省下达的保障性住房建设任务。实现扶贫"双到"三年目标，贫困村脱贫率达98%。

　　社会事业全面进步。教育创强工作稳步推进，普通高考再创佳绩、总上线率80.7%，高中阶段毛入学率86.7%；高、中职教育加快发展；中小学校舍安全工程全面完成。2012年本级财政教育支出18.1亿元，占一般预算支出的21.8%，超出省下达任务1.2个百分点。医疗卫生体制改革不断深化，基本药物制度实现全覆盖，县级公立医院改革试点正式启动。公共文化基础设施不断完善，新建文化站11个、文化室284个；文艺创作成果丰硕，西秦戏《留取丹心照汗青》获第八届省精神文明建设"五个一工程"奖，陆丰皮影戏摘取我市首个国际艺术奖项。殡改工作强势扭转落后局面，全市5个县（区）、51个镇（街道、场）的火化率达到100%。十件惠民实事基本完成。体育、计生、统计、侨务、档案、修志、国防、人防、气象、防震、口岸、港澳台、民族宗教、工青妇等工作取得新成绩。

　　平安建设深入推进。社会大局和谐稳定，刑事立案数、每万人刑事发案数均排在全省末位，十八大期间实现进京"零上访"目标；食品药品安全、安全生产形势平稳；市应急平台正式启用，妥善处置韩国籍货轮"雅典娜"沉没事故，成功破解了海上危化品打捞"世界级难题"；劳动关系总体稳定；"三打两建"工作成效显著。

　　（四）民主法制与政府自身建设不断加强。

　　自觉接受市人大及其常委会和市政协的监督，人大议案和建议、政协提案办复率100%。"六五"普法全面推进。审计、监察、法制工作不断加强。行政审批制度改革进一步深化，事业单位分类改革基本完成。"责任、督查、奖罚"三大机制得到落实，市政务服务中心进一步完善，100%涉企行政审批单位进入中心，0A系统建成运行，效能监察、党风廉政建设进一步加强，政府效率和服务水平有所提升。

　　各位代表，2012年取得的成绩，来之不易。这是省委、省政府及市委正确领导的结果，是市人大及其常委会和市政协大力支持的结果，是全市人民团结奋斗的结果，也是海内外各界人士关心支持的结果。在此，我代表市人民政府，向关心支持汕尾发展、倾注心血、付出辛劳的各界人士和广大市民表示崇高的敬意！向长期关心支持我市改革发展的港澳台同胞、海外侨胞表示衷心的感谢！

　　在肯定成绩的同时，我们也要看到，我市经济社会发展还存在不少压力和困难：一是经济规模小，发展质量不高。GDP虽然连续8年高位增长，但总量仅占全省的1.1%左右；财政收入仅占全省的0.65%，且非税收入比重偏高，财政收支矛盾突出；产业化程度较低，规上工业总量较小，第三产业发展滞后，自主创新能力处于低水平。二是城市化水平低，市区辐射力弱。市区（包括市直和城区）GDP仅占全市经济总量的23.8%，公共财政预算收入仅占全市的34.4%；市区扩容提质进展慢，交通基础设施和市政公共服务配套不够完善，城市管理水平亟待提高。三是社会事业欠账多，管理压力大。基本公共服务均等化水平不高，城乡区域发展差距较大，部分群众生活仍然困难，就业还不够充分；尤其是教育、计生、卫生、殡改、社会保障、征地拆迁、信访维稳、社会治安等工作尚待加强。四是资源环境管理水平低、任务重。土地、资金、人才、技术等要素制约愈加明显，环保意识仍需加强，生态保护、节能减排，土地、矿产、环境管理等问题仍需下大力气解决。五是政府职能转变尚未到位，干部执行力不够强。一些干部发展意识淡薄，安于现状，抓工作的拼劲和狠劲不足；行政效能和服务水平与人民群众的期望还有一定差距。对此，我们必须高度重视，采取有效措施加以解决。

　　二、2013年工作安排

　　当前，我市正处于科学跨越发展、建设幸福汕尾的黄金时期。纵观国内外形势，我市面临难得的发展机遇，随着贯彻落实党的十八大精神的不断深入，省"八个行动计划"全面实施，一批重点项目相继落户建设，特别是厦深铁路即将建成通车，汕尾融入珠三角的区位、资源优势日渐凸显，产业集群初具雏形，汕尾加快发展条件已经成熟。我们一定要坚定信心，把握机遇，脚踏实地，埋头苦干，奋力开创经济社会全面协调可持续发展新局面。

　　2013年政府工作的总体要求是：以邓小平理论、"三个代表"重要思想和科学发展观为指导，全面贯彻落实党的十八大、中央经济工作会议、习近平总书记视察广东重要讲话和省委十一届二次全会、市委六届二次全会精神，以提高经济增长质量和效益为中心，进一步增强加快发展的意识，深入实施"四大战略"，大力推进"八大转型"，强化创新驱动，深化改革开放，打造软硬环境，促进绿色发展，着力保障和改善民生，增强经济发展的内生活力和动力，加快建设宜居宜业宜憩的现代化滨海新城，为推动科学跨越发展、建设幸福汕尾，争当促进区域协调发展排头兵而努力奋斗。

　　2013年的主要预期目标是：生产总值增长13.5%，人均生产总值增长12.5%；固定资产投资增长30%；规模以上工业增加值增长28%；社会消费品零售总额增长15%；外贸出口总额增长15%；公共财政预算收入增长20%；城镇居民人均可支配收入增长12%；农村居民人均纯收入增长12%；城镇登记失业率控制在3%以内；节能减排约束性指标完成省下达的年度计划。为此，必须着力抓好八项工作：

　　（一）着力构建现代产业体系。

　　要把推进产业转型升级作为主攻方向，深入实施"海岸经济"战略，加快构建现代产业体系。

　　深入推进区域合作。加快区域协调发展，积极主动承接珠三角产业、资金、技术转移。举全市之力，加快深汕特别合作区建设。充分利用深圳在产业、人才、技术、体制等方面的优势和在开发建设中的主导作用，全力支持深汕特别合作区履行主体职责，在完善体制机制、规划编制、土地整备、基础设施等工作的基础上，集中精力抓好招商引资和项目落户，争取早日上马、早日建成、早见成效，努力打造一流示范品牌园区。加强与珠三角内环其他五市合作，办好第二届内环六市论坛，促进与周边地区开放优势互补，培育带动区域发展新高地。推进省级开发区整合改造，加快各县（市、区）工业园开发建设，力争每个园区有3-5个重大产业龙头项目落户。

　　加快重点项目建设。今年安排市重点建设项目42项，年度计划投资89.6亿元。重点抓好陆丰核电、华润海丰电厂、陆丰甲湖湾电厂等投资超100亿元电力能源项目，信利4.5代AM-OLED、腾讯云计算等一批投资超30亿元电子信息项目，内湖农产品批发市场、好日子城市综合体等一批超5亿元商贸旅游项目上马建设。

　　打造科技创新服务平台。完善科技服务体系，建设产学研示范基地和战略联盟，加快战略性新兴产业的产品开发。搭建创新平台，抓好深圳大学光电产业孵化园起步建设和省级高新技术产业开发区申报。推进研发机构和专业镇中小微企业公共服务平台建设，重点抓好"海洋产业研究院"、"广东华南科技资本研究院汕尾分院"和"检验检疫公共技术检测平台"建设。实施"质量强市"战略，鼓励企业争创名牌和参与行业标准制（修）订。

　　培育战略性新兴产业。加快培育发展电子信息、新能源、生物医药等战略性新兴产业。依托信利4.5代AM-OLED新型显示器件的研发及产业化，加快三维显示等新型技术及下一代视频技术研发，培育国家级电子信息产业基地，推动电子信息产业升级。大力发展软件服务、网络增值服务等信息服务业。积极发展风能、太阳能等新能源产业。

　　大力发展旅游业。完善旅游发展规划，用好省扶持资金，全力推动红海湾滨海旅游产业园区、海丽高尔夫球场海燕半岛酒店和长沙湾度假城等旅游项目上马建设。完善旅游基础配套设施，加快景区景点改造升级，抓好凤山妈祖4A级景区创建工作，打造国内知名旅游目的地。

　　力促商贸流通业发展。加快商贸物流网点规划建设，优化商贸流通业布局，打造区域性物流中心。重点抓好华德国际商贸物流城、中国（汕尾）珠宝交易中心、火车站枢纽物流中心、粤东水产物流中心等项目建设。继续抓好"万村千乡"市场工程、农贸市场标准化改造工程、"农超对接"和平价商场建设，推进红星美凯龙、岁宝百货等大型商贸项目落地投产，促进商贸物流业升级发展。

　　推动传统产业改造升级。大力扶持传统优势产业，延伸产业链条，提升产业层次，加快转型升级。打造"公平服装"、"可塘珠宝"、"碣石圣诞品"、"鹅埠鞋业"、"甲子五金"和"梅陇金银首饰"等区域特色品牌，形成一批产业集聚度较高、创新能力较强的中心镇和专业镇。

　　加快外经贸转型发展。强化产业政策引导，创新招商引资机制，全面铺开专业化招商，瞄准世界500强、国际知名企业开展定向招商、产业招商、以商引商。大力发展服务贸易和服务外包。稳定出口，扩大进口，积极转变外贸发展方式，加快培育外贸转型升级示范基地、加工贸易转型升级示范企业。

　　（二）着力推进农业产业化。

　　解决好"三农"问题是我市加快发展的重中之重。要把加快农业产业化发展作为突破口，力促农业增效，农民增收，农村稳定。

　　加强农业基础设施建设。加快"百里海堤爱海工程"建设，抓好百宗治洪治涝保安工程、农田水利工程、18万亩高标准基本农田建设工程、村村通自来水工程和农村饮水安全工程。用好省扶持资金，加快陆丰市村村通自来水工程示范县建设。

　　提升农业产业化水平。发展农业产业化经营是改变我市农村地区自给自足传统农业经济、扭转农业弱质低效局面的根本出路。要以现代农业基地为示范带动，着力培育新型经营主体，做强农业龙头企业，做实农民专业合作组织，培育园艺优势产业，实施原生态菜篮子工程，发展特色效益农业，提高农民组织化程度和农业产业化水平。坚持科技兴农，加快农业科技园区建设，提高农业技术装备水平和农业科技贡献率。加强农产品品牌建设和质量认证。创造更好条件，鼓励乡贤回归投资农业产业化项目。以省建设国家海洋经济综合试验区为契机，大力发展深外海捕捞和水产品深加工业，抓紧推进汕尾中心渔港和遮浪渔港建设，加快南国渔都品牌创建、海上养殖园区申报，做大海洋经济支柱产业。

　　多渠道增加农民收入。落实中央和省强农惠农富农政策，鼓励农民优化种养结构，加快农村二三产业发展，努力增加农民转移性收入、生产性收入、经营性收入、工资性收入。启动新一轮扶贫开发"双到"工作，完善扶贫开发长效机制。

　　（三）着力扶持企业做大做强。

　　企业是经济社会发展的微观基础。必须为企业营造良好的发展环境，发挥企业作为市场经济和转型升级的主体作用，提升企业竞争力。

　　加大企业扶持力度，优化涉企行政服务。健全面向中小企业的服务体系，打造民营企业和中小企业融资、综合审批、用工及人力资源、商会行业协会"四大服务平台"。建立完善企业联合会与涉企职能部门的沟通机制，搭建涉企信息和惠企政策宣传发布平台。协调土地、劳动力、财力等资源要素向优势、骨干企业倾斜，积极扶持企业上市，力促企业做大做强。积极组织企业参加招商洽谈会。

　　着力盘活存量、引进增量。激活存量促升级，优化做大现有企业，培育一批具有核心竞争力、经营上规模的骨干企业。大力引进行业龙头企业和优质中小企业，力争以量的增大，促进质的飞跃。

　　营造重企爱企氛围，更好发挥企业家作用。企业家是全市经济发展的稀缺资源，要十分珍惜企业家的作用。维护民营企业和民营企业家的合法权益，形成全社会尊重企业家、支持企业家的营商环境。深入实施"反哺工程"，鼓励乡贤回归创业。支持企业培育符合时代精神、独具特色的优秀企业文化。加强行业职业道德建设，引导企业家恪守道德底线、诚信守法经营、勇担社会责任。

　　（四）着力推进新型城镇化。

　　提高城镇化质量，要突出抓好中心市区扩容提质，以县城建设为龙头，以中心镇建设为抓手，全面实施"宜居城乡"战略，增强城镇发展竞争力。

　　加快交通等城市基础设施建设。全面实施《交通综合规划》，加快推进江西寻乌至汕尾、龙川至汕尾高速公路及广州至汕尾、龙川至汕尾铁路前期工作，全线开工建设潮莞高速汕尾段。确保厦深铁路汕尾段按期建成投入运营。改造建设一批国省道和农村公路项目，加快启动珠东沿海快速通道建设，构建区域快速便捷交通网络。抓紧完成汕尾港总体规划报批工作，开展汕尾港（一期）10万吨级码头建设前期工作，抓好汕尾港航道疏浚工程。

　　加快中心市区扩容提质。拓展中心市区发展空间，增强集聚辐射功能。突出规划龙头引领作用，启动编制《汕尾市环品清湖规划》及市区绿道网和慢行系统等专项规划，完善城市建设规划，提高控规覆盖率。投入10亿元，加快市区新湖大道、工业大道、环品清湖道路、汕尾火车站站前广场及周边道路、汕尾大道改造工程、埔边至市区公路改造工程、市区污水处理厂管网工程以及LED路灯改造和推广应用等基础设施及配套市政建设，完善市政电网、道路网、绿道网、地下管网体系。加快"智慧汕尾"、"无线城市"建设，提高信息化应用水平。开展"城市管理年"活动，强化精细管理，深入开展市容市貌、环境卫生、道路交通等集中整治行动，营造优美整洁城市环境。加强市民公共道德教育，规范城市公共秩序，提升城市文明程度。

　　提升城镇化水平。优化中心市区功能定位和产业布局，走集约、智能、绿色、低碳的新型城镇化发展道路。加快发展中心镇，增强人口和产业集聚能力、综合承载力。完善城镇落户政策，推动农村富余劳动力向城镇转移就业落户。以推进农业产业集中发展、农民集中居住、土地集中经营为重点，加快农村社区建设，提高农村地区管理水平。加强农村金融服务工作。加快城市基础设施向农村地区延伸和覆盖，深入开展农村环境卫生整洁行动和名镇名村示范点创建工作，大力改善农村生态、生活环境，力争用两至三年时间，每个行政村建设一个公益性生态公园或休闲广场，努力创建幸福村居。加快县（市、区）及建制镇总体规划修编，抓好村镇规划编制工作，力争2015年前完成全部建制镇规划，完成70%以上村庄规划。

　　（五）着力推进生态文明建设。

　　建设生态文明关系人民福祉、关乎汕尾未来。要坚持在保护中发展，在发展中保护，强化生态资源环境管理，加强节能减排和污染防治，加快绿色、循环、低碳发展，实现经济效益、社会效益、生态效益相统一，形成人人爱护生态环境的浓厚氛围，建设美丽汕尾。

　　加强资源管理。加强对敏感地带及海岸线、湿地、森林、矿山等重要资源的保护和管理。实施最严格的水资源管理制度，划定乡镇以上饮用水源保护区，开展公平水库（含干渠)、 螺河等水资源保护规划编制工作。实施最严格的耕地保护制度，落实基本农田保护经济补偿制度。抓好土地利用总体规划落实，实施最严格的节约集约用地管理。依法垄断土地一级开发。加大土地储备力度，加快闲置土地清理处置，全力推进火车站片区、金町湾片区、红草工业区、碧桂园二期等重点用地征地工作，力争实现市区盘活存量建设用地1500亩、储备土地3000亩目标。改进征地管理，完善征地补偿措施，统筹考虑被征地农民的长期生计，把更多的利益留给基层群众。加快保障性住房建设和"三旧"改造，因地制宜改造城中村，整治空心村。

　　推进生态工程建设。以启动市环境监测监控中心建设为抓手，完善环境监测预警体系。加快市生活垃圾无害化处理中心建设。抓好以品清湖为重点的港湾整治，加强奎山河、龙津河、螺河等主要河流保护。以推进新一轮绿化广东大行动为契机，抓好深汕高速汕尾段70公里生态景观林带、11万亩防护林工程和26万亩森林碳汇重点生态工程建设，加强市区山体的有效保护和规划利用，巩固创园成果。加快建设陆河生态文明县。

　　（六）着力深化重点领域和关键环节的改革。

　　我市作为欠发达地区，经济社会发展欠账多、任务重，必须深化市场化改革,向市场化改革要资源、要效益、要质量、要活力。

　　深化经济体制改革。加大财税体制改革力度，健全财税保障机制，推进营业税改征增值税试点改革，建立完善税费一体化管理机制。深化投融资体制改革，引入银行、证券、保险机构来我市开设分支机构，发展小额贷款公司和融资性担保机构，拓宽中小微企业融资渠道。深化农村信用社改革，组建农村商业银行。积极利用市场资本，鼓励和引导更多民间资本参与基础设施、市政公用等领域建设。尽快落实10亿元贷款，确保重点建设项目、民生工程资金需求。完善土地、人才、技术、信息等要素市场。

　　深化行政体制改革。加快推进行政审批标准化建设，市县精简行政审批事项达到60%。完成县级大部门制和行政体制改革。深化富县强镇、简政强镇事权和事业单位改革。推进市区事权改革，理顺解决市与城区之间体制机制不顺、职责关系不清、管理服务职能不到位等问题。深化医疗卫生、文化教育、社会保障等民生事业体制改革，加快推进城市管理、造林绿化等领域的市场化。

　　抓好社会管理改革。培育发展和规范管理社会组织，支持、引导其参与社会公共服务管理。完善政府购买社会服务制度。推进基层社会管理体制改革，加强农村公共服务规范化建设，健全基层群众自治机制，推进社会协同善治。严厉打击欺行霸市、制假售假、商业贿赂等违法犯罪行为，全面推进"两建"工作。

　　（七）着力保障和改善民生。

　　保障和改善民生是一切工作的出发点和落脚点。要坚持民生优先，加大投入，大力推进基本公共服务均等化，让改革发展成果惠及人民群众。

　　抓好就业和社会保障工作。实施就业优先战略，加大高校毕业生、异地务工人员、就业困难群体和退役军人的就业帮扶力度，加快社区公共就业服务平台联网，促进充分就业和高质量就业。推进社保扩面征缴。加快城乡居民医疗保险基金市级统筹，实现省内异地就医多向联网即时结算和职工医保关系转移接续。创新商业机构经办医保管理服务新模式。加快社保卡"一卡通"和"金保"工程建设。拓宽社会保障基金筹资渠道，加强基金监管。

　　加快发展教育事业。全面实施"教育创强"工作，力争新增省教育强镇19个。巩固提高"普高"的质量和水平，确保顺利通过省复评。加快乡镇中心幼儿园、村级幼儿园建设。促进义务教育均衡发展。加强教师继续教育和培训，全面提高师德水平和综合素质。认真实施外来教师"温暖工程"，关心教师工作和生活，营造尊师重教的浓厚氛围。大力发展职业教育，办好高等职业教育，加快中职学校建设。继续办好教育园区。加快教育信息化"三通两平台"建设，投入1.8亿元，力争两年全市公办中小学实现课室多媒体电教平台全覆盖。支持特殊教育和民办教育发展。

　　加快发展文化事业。广泛开展群众性文明创建活动。大力推进公共文化设施建设，加快建设中国（汕尾）民间文化艺术馆、市图书馆(扩建)和"数字影院"以及镇（街道）文化站、村级文化室（广场）。继续实施文化惠民工程。提高文化产业在经济总量中的比重。加强文化遗产保护和开发利用。加强文艺精品创作和对外文化交流。

　　加快发展卫生医疗事业。完善城乡卫生均等化服务体系，抓好省卫生城市创建工作。推动市第一人民医院和第二人民医院创三甲医院，推进公立医院改革试点工作，加大投入，加快医疗卫生信息平台建设，引进社会资本，多措并举，促进医疗水平提升。大力发展中医药事业，加快南方中医院建设。探索平价医院试点模式。加强食品药品监管。广泛开展全民健身运动。

　　大力创建平安汕尾。健全社会治安立体防控体系，推行 "科技强警"战略，依法严厉打击各类刑事犯罪活动，继续抓好陆丰社会治安突出问题和禁毒整治，加强反走私工作。完善综治信访维稳体系，加快人民来访接待大厅、网上信访大厅建设，探索解决重访问题的有效途径，妥善处理好人民群众的合理诉求。全面落实安全生产"一岗双责"制度，坚决防范和遏制重特大事故发生。健全自然灾害、事故灾难、公共卫生事件、社会安全处置机制，提高应急管理工作水平。

　　推进殡改、人口与计生工作。继续巩固和扩大殡改工作成果，全面治理乱埋乱葬坟墓，加快乡镇公益性公墓山（骨灰楼）等殡葬设施建设，确保火化率达到100%。创新人口服务与管理机制，大力实施优生健康惠民工程，建立健全优质服务保障机制，拓展服务内容，提升服务水平，为育龄群众创造温馨舒适的服务环境，切实稳定低生育水平。

　　同时，注重解决民生突出问题，重点办好十件民生实事。

　　（八）着力推进政府自身建设。

　　做好今年工作，是对政府施政能力的考验。要切实改进作风、真抓实干，努力提高政府执行力和服务水平。

　　提高政府执行力。全面开展"政府执行力建设年"活动，坚持从领导做起，从市政府办公室等市直部门做起，落实绩效考核，切实提高工作执行力；启动流程再造，确保及时办结；完善社会评价机制，落实"责任、督查、奖罚"三大机制，坚持推进重大项目、重大活动、民生实事等重大工作以及市委、市政府重大决策倒逼机制，坚决扭转有令不行、执行不力、效率不高、工作平庸的作风，营造真抓实干、执行有力、奋发有为的创业氛围。

　　提高政府服务水平。加强部门协作，整合政务服务资源，建设综合政务服务体系。完善政务服务中心，加快电子政务建设，抓紧建成网上办事大厅，与省级网上办事大厅无缝对接，实行网上审批、网上办事。加快多层次、全方位的服务网络建设。开通12345市民服务热线。建设微博等各种问政平台，为群众提供更多参政议政渠道。深入推进政务公开，充分利用媒体、微博等信息手段做好政府信息公开工作。加强教育培训，提高公务员整体素质和服务水平。

　　建设民主法治廉洁政府。自觉接受人大法律监督和政协民主监督，自觉接受舆论监督和群众监督。积极推进"六五"普法和法治政府建设，加强执法公开、政务公开、行政复议和行政应诉工作，提高依法行政水平。健全公众参与、专家论证和政府决定相结合的行政决策机制，提高决策的透明度和科学水平。加强廉洁从政教育和廉政文化建设。落实廉洁从政制度，推进廉政风险防控。认真贯彻执行中央改进工作作风、密切联系群众"八项规定"，坚持厉行节约、勤俭办事，严格控制"三公"消费和一般性支出。

　　各位代表！实现今年的各项目标任务要靠实干。让我们更加紧密地团结在以习近平同志为核心的党中央周围，在市委的坚强领导下，开拓创新，埋头苦干，为加快汕尾经济社会发展而努力奋斗!
